# Pediatric stroke in an African country

**DOI:** 10.4103/1817-1745.66676

**Published:** 2010

**Authors:** Julius Alexander Ogeng’o, Beda O. Olabu, Anne N. Mburu, Simeon R. Sinkeet

**Affiliations:** Department of Human Anatomy, University of Nairobi, PO Box 30197-00100, Nairobi, Kenya

**Keywords:** African, risk factors, pediatric stroke

## Abstract

**Background:**

The pattern of pediatric stroke displays ethnic and geographical variations. There are few reports from black Sub-Saharan Africa, although relevant data are important in prevention, clinical diagnosis, treatment and prognostication.

**Aim:**

To describe subtypes, risk factors, localization, age and gender distribution of pediatric stroke in the black Kenyan population.

**Study Design and Setting:**

Retrospective cross-sectional study in a single regional referral and teaching hospital.

**Statistical Analysis:**

Data were analyzed by SPSS version 13.0 for Windows and presented in tables and bar and pie charts.

**Materials and Methods:**

The study was performed at the Kenyatta National Hospital, a level-6 regional referral health facility with an annual pediatric in-patient turnover of about 40,000 patients. Files of patients aged 1 month to 18 years over a period of 5 years were analyzed for stroke subtypes, localization, risk factors, age and sex distribution. Only those files with complete information were included.

**Results:**

Thirty-two of the 712 stroke patients (4.5%) were pediatric. The male:female ratio was 1.7:1. Ischemic stroke comprised 56.3% (n = 18). Mean age was 7.7 years (range, 1.5–18 years). The most common sites were cortical (51%), lacunar (41%) and brain stem (8%). The most common risk factors were connective tissue disorders (28.1%), heart disease (25%), human immunodeficiency virus (9.4%) and infection (9.4%).

**Conclusion:**

Pediatric stroke is not uncommon in the Kenyan population. The risk factor profile comprising connective tissue disorders and infection differs from that reported in other populations, inviting large community-based studies.

## Introduction

Pediatric stroke is an important cause of mortality and morbidity.[1,2] Its pattern displays geographical and ethnic variations, probably related to differences in risk factors.[3,4] Because of the different demographic characteristics and higher prevalence of predisposing factors, stroke incidence in Sub-Saharan Africa may be higher than in developed countries.[5] Data on risk factors, age, gender distribution and outcome are important for identifying significant areas of future treatment and prevention.[1] Reports from Sub-Saharan African countries, however, are scarce, and altogether absent for Kenya. This study reports the characteristics of pediatric stroke in this Sub-Saharan African country.

## Materials and Methods

This was a retrospective study performed at the Kenyatta National Hospital (KNH), a level-6 Eastern and Central African regional referral and teaching hospital in Nairobi, Kenya, with a total pediatric patient population of about 40,000 per year. Ethical approval for the study was granted by the Kenyatta National Hospital/University of Nairobi Ethics and Research Committee (KNH/UoN-ERC). Files of patients aged between 1 month and 18 years with a diagnosis of stroke, seen between January 2004 and December 2008, were retrieved from the hospital registry. Patients were categorized into male and female, and each was sex divided into five year age groups. Only files with complete data were included. Each file was analyzed for presentation, mode of diagnosis, stroke subtype, localization, risk factors and outcome. These data were analyzed using SPSS version 13.0 (Chicago, IL, USA) for Windows and presented by means of tables and bar and pie charts.

## Results

**Figure 1:**
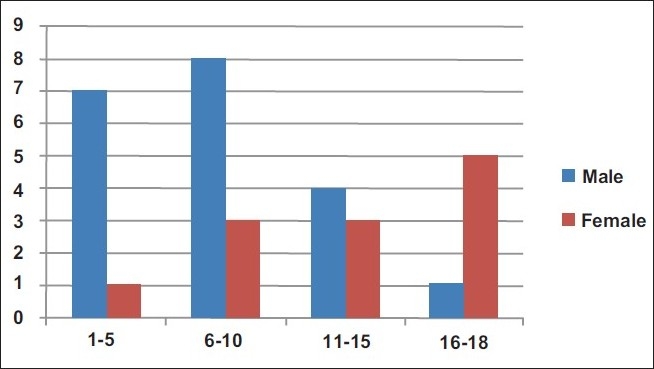
Age and gender distribution of stroke

Thirty-two cases of pediatric stroke out of 712 (4.5%) were retrieved in a pediatric inpatient population of about 200,000 over the study period. Twenty of these (62.5%) were males while 12 (37.5%) were females. Male predominance reduced with age such that by 16–18 years, females exceeded males. Mean age was 7.7 years, with the peak occurring in the 6–10-year age group [Figure 1].

Ischemic and hemorrhagic strokes comprised 18 (56.3%) and 14 (43.7%), respectively. Of these, 17 (53.1%) were cortical types, distributed as eight frontoparietal, four parietal, three temporal, one occipital and one frontal. The remaining 15 cases (46.9%) were subcortical types, located in the basal ganglia (5), internal capsule (4), thalamus (4) and brain stem (2).

The most common modes of presentation in younger children (below 5 years) were focal weakness (2), seizures (2), altered mental status (1) and mixed symptoms (3). In older children, it was mixed symptoms (11), hemiparesis (5), altered mental status (3) and other focal neurologic signs, such as aphasia and visual disturbance (5). Nine cases (28.1%) were diagnosed clinically while 23 (71.9%) were confirmed by computed tomography (CT) scan.

The major risk/comorbid factors were connective tissue disorders (28.1%), congenital heart disease (25.0%), human immunodeficiency virus (HIV) infection (9.4%), other infections (9.4%) and sickle cell disease (6.3%). In two cases (6.3%), there were multiple comorbid conditions while in five cases (15.6%) no risk/comorbidity was determined [Table 1]. Thirteen patients (40.6%) recovered fully within 3 months, 17 (53.1%) developed neurological deficits while two (6.3%) died.

**Table 1:** Distribution of risk factors

Risk factor	Frequency (%)
Connective tissue disorder	9 (28.1)
Heart disease	8 (25)
HIV	3 (9.4)
Infection	3 (9.4)
Sickle cell disease	2 (6.3)
Multiple	2 (6.3)
Undetermined	5 (15.6)
Total	32 (100)

## Discussion

The estimated prevalence of 16 per 100,000 is higher than the 1.29–13.0 per 100,000 reported in other populations.[6] This suggests that pediatric stroke is not uncommon in the Kenyan population.

The mean age of 7.7 years observed in the present study is comparable to 7.7 years reported for an American population,[1] but higher than 5.6 years among the Chinese.[3] These figures are at variance with reports of the 2009 heart disease and stroke statistics that there is a peak in the perinatal period. This suggests that age-related risk factors differ substantially between populations. Further, observations of the current study reveal, concordant with other studies, that pediatric stroke shows male predominance.[7] However, after puberty, this difference reduces, such that by 16–18 years of age, there is a female predominance. The latter appears concordant with reports from some Asian communities that stroke also occurs more commonly among girls.[8,9] The reasons for this are unclear, but are probably related to the profile of risk factors even though genetic X-linked disorders have been implicated.[7]

As in the literature reports,[10,11] ischemic stroke was more common than hemorrhagic stroke. Isolated reports have reported that hemorrhagic and ischemic strokes are equally common.[12] These suggest that the stroke subtypes vary between populations, probably also depending on the distribution of risk factors.

The most common cause of ischemic stroke among black children is reported to be sickle cell disease.[11] Observations of the present study reveal a wide variety of comorbid/risk factors, the leading of which was connective tissue disorders. The profile of implicated factors shows notable differences from those reported in the literature [Table 2].

**Table 2:** Predominant risk factors reported in the literature

Author	Population	Sample size	Predominant risk factors (%)
Chung and Wong, 2004[3]	Chinese	50	Congenital heart disease (30), hematological disease (28), vascular disease (26)
Giroud *et al*., 1993[13]	French	28	Infection (22.6), trauma (22.6), heart disease (19.4)
Bowen *et al*., 2005[14]	American	27	Arterial dissection (19), coagulopathy (15), embolism (15)
Riikonen and Santavuori[15]	Finnish	44	Infection (34), migrane and thrombotic (13.6), mitochondrial disease (4.5)
Ham *et al*., 2009[16]	Singaporean	26	Collagen vascular disorders (33.3), metabolic disorders (26.7), vascular arteriopathy (15.4)
Current study	Kenyan	32	Connective tissue disorders (28.1), heart disease (25), HIV (9.4), infection (9.4)

These studies reveal that risk factors vary between countries and ethnic groups, suggesting that environmental factors interact with genetic predisposition to determine the overall risk. This implies that there is a need for the evaluation of population-specific factors. A notable observation of the current study is the high prevalence of connective tissue disorders comparable to the Singaporean population.[16] The connective tissue disorders implicated in stroke[17] are genetically determined. This suggests that a large proportion of pediatric stroke cases have a genetic basis, and invites search for these factors. Direct genetic contribution in stroke acts in combination with environmental factors or via other epistatic factors, i.e. gene–gene or gene–environment.[18]

HIV was implicated in 9.4% of the cases in the present study. HIV/AIDS increases the risk of both ischemic and hemorrhagic stroke after correcting for other cardiovascular risk factors.[19,20] This implies that HIV infection should be included in the differential diagnosis of pediatric stroke[21] and that better control of HIV may constitute a useful control measure for stroke.[19] Other acute or chronic viral and bacterial infections contribute to increased risk of stroke by elevating the risk of embolism.[22,23]

Observations of the current study reveal that infection was implicated in 9.4% of the cases. This appears to be commensurate with reports that infectious burden index is associated with increased risk of all strokes.[24] In over 15% of the cases, a risk factor was not determined, as is reported in the literature.[25] This may be due to the limited investigations or lack of suspicion, but could also be due to nonconventional risk factors hitherto unexplored. These include socioeconomic status, environmental toxicity, genetic predisposition and ethnocultural factors.[25] This invites more concerted effort in the search for risk factors.

## Conclusion

Pediatric stroke is not uncommon in the Kenyan population. Its characteristics resemble those reported for Caucasian and Asian populations. This risk factor profile comprising connective tissue disorders and infection differ from those reported for other populations, inviting large community-based studies.

## References

[1] Lynch JK, Hirtz DG, De Veber G, Nelson KB (2002). Report of the National Institute of Neurological Disorders and Stroke Workshop on perinatal and childhood stroke. Paediatrics.

[2] Zahuranec DB, Brown DL, Lisabeth LD, Morgensten LB (2005). Is it time for a large collaborative study of paediatric stroke?. Stroke.

[3] Chung B, Wong V (2004). Paediatric stroke among Hong Kong Chinese subjects. Paediatrics.

[4] Fullerton HJ, Elkins JS, Johnstone SC (2004). Paediatric stroke belt: Geographic variation in stroke mortality in US children. Stroke.

[5] Ganesan V (2007). Arterial ischaemic stroke in childhood. Ann Indian Acad Neurol.

[6] Kirkham F, Sebire G, Steilin M, Strater R (2004). Arterial ischaemic stroke in children: Review of literature and strategies for future stroke studies. Thromb Haemost.

[7] Golomb MR, Fullerton HJ, Nowak-Gottl U, Deveber G (2009). International Pediatric Stroke Study Group. Male predominance in childhood ischaemic stroke: Findings from the International Paediatric stroke study. Stroke.

[8] Ham EH, Tay KH, Low PS (2009). Factors predictive of outcome in childhood stroke in an Asian population. Ann Acad Med Singapore.

[9] Siddiqui TS, Rehman A, Ahmed B (2006). Aetiology of stroke and hemiplegia in children presenting at Ayub Teaching Hospital, Abbattabad. J Ayub Med Coll Abbattabad.

[10] Wang JJ, Shi KL, Li JW, Jiang LQ, Caspi O, Fang F (2009). Risk factors for arterial ischaemic and Haemorrhagic stroke in childhood. Paediatr Neurol.

[11] Lloyd-Jones D, Adams R, Carnethon M, De Simone G, Ferguson TB, Flegal K (2009). Heart disease and stroke statistic 2009 update: A report from the American Heart Association Statistics committee and stroke statistics subcommittee. Circulation.

[12] Broderick J, Talbot GT, Prenger E, Leach A, Brott T (1993). Stroke in children within a major metropolitan area: The surprising importance of intracerebral haemmorhage. J Child Neurol.

[13] Giroud M, Lemesle M, Madinier G, Manceau E, Oseby GV, Dumas R (1997). Stroke in the children under 16 years of age: Clinical and aetiological difference with adults. Acta Neurol Scand.

[14] Bowen MD, Burack CR, Barao TF (2005). Childhood ischaemic stroke in a non urban population. J Child Neurol.

[15] Riikonen R, Santavuori P (1994). Hereditary and acquired risk factors for childhood stroke. Neuropaediatrics.

[16] Tham EH, Tay SK, Low PS (2009). Factors predictive of outcome in childhood stroke in an Asian population. Ann Acad Med Singapore.

[17] Tonarelli SB, Benavente O (2005). Heritbale connective tissue disorders and stroke. Semin Cardiovasc Dis Stroke.

[18] Hankey GJ (2006). Potential risk factors for ischaemic stroke: What is their potential?. Stroke.

[19] Dobbs MR, Berger JR (2009). Stroke in HIV infection and AIDS. Expert Rev Cardiovasc Ther.

[20] Qureshi AI (2005). HIV infection and stroke: If not protein S deficiency, then what explain the relationship?. J Neurosurg Psychiatry.

[21] Narayan P, Samuels OB, Barrow DL (2002). Stroke and paediatric human immunofdeficiency virus infection: Case report and review of literature. Paediatr Neurosurg.

[22] Grau AJ, Buggle F, Becher H, Zimmerman F, Spiel M, Fent T (1998). Recent bacterial and viral infection is a risk factor for cerebrovascular ischaemia: Clinical and biochemical studies. Neurology.

[23] Lindsberg PJ, Grau AJ (2003). Inflamation and infections as risk factors for ischaemic stroke. Stroke.

[24] Elkind MS, Ramakrishnan P, Moon YP, Boden-Albala B, Liu KM, Spitalnik SL (2010). Infectious burden and Risk of stroke: The northern Manhattan study. Arch Neurol.

[25] Fullerton HJ, Elkins JS, Johnstone SC (2004). Paediatric Stroke Belt: geographic variation in stroke mortality in US children. Stroke.

